# Role of angiogenic signaling through placental growth factor in white matter injury and cognitive decline

**DOI:** 10.4103/NRR.NRR-D-25-00454

**Published:** 2026-01-27

**Authors:** Madhavi Akella, Parissa Irom, Kyle C. Kern, Jason D. Hinman

**Affiliations:** Department of Neurology, David Geffen School of Medicine, University of California Los Angeles, Los Angeles, CA, USA

Vascular contributions to cognitive impairment and dementia (VCID) remain a significant public health concern. Age-associated disruptions in cerebral blood flow and brain vascular permeability resulting from cerebral small vessel disease (CSVD) are the main drivers of VCID. There is a stark absence of pharmacological treatments specific to CSVD, in part due to challenges in diagnostic accuracy for CSVD pathology during the prodromal phase before clinical dementia. Magnetic resonance imaging (MRI) is the benchmark method for detecting cerebrovascular pathologies, which predominate in subcortical white matter regions. Advanced MRI techniques, including diffusion tensor imaging (DTI), which have higher diagnostic accuracy for prodromal CSVD are not routinely acquired in clinical scans. Highly sensitive and accurate blood-based biomarkers that inform the pathogenic cascades associated with CSVD and VCID offer a minimally invasive means of identifying individuals who are at risk for VCID.

White matter hyperintensities (WMH) observed on MRI are an indicator of CSVD and are proposed to arise from chronic hypoperfusion, endothelial dysfunction, blood–brain barrier disruption, and extravasation of fluid and inflammatory mediators to the extravascular space. These mechanisms contribute to progressive white matter injury and ultimately lead to cognitive decline via a disconnection syndrome. One approach to improving biomarker specificity for CSVD is to integrate and utilize multiple blood-based biomarkers, such as inflammatory (e.g., C-reactive protein and tumor necrosis factor-alpha), white matter injury (e.g., neurofilament light chain and glial fibrillary acidic protein), and endothelial markers reflective of underlying vascular injury. A key objective in biomarker development is ensuring that markers exhibit strong specificity to the proposed mechanisms of CSVD to be clinically useful. This is because VCID has overlapping pathophysiology with other neurodegenerative conditions, including Alzheimer’s disease, making differentiation challenging. Amongst potential endothelial candidate biomarkers is placental growth factor (PIGF), an angiogenic signaling molecule that plays a critical role in angiogenesis and vascular remodeling. This perspective focuses on the emerging role of PlGF as a diagnostic and susceptibility/risk biomarker for VCID that reflects CSVD pathology.

**Placental growth factor:** Placental growth factor is a 29 kDa dimeric glycoprotein that is a member of the vascular endothelial growth factor superfamily of angiogenic signaling molecules. It is secreted by endothelial cells and macrophages in response to inflammatory stimuli. The vascular endothelial growth factor receptor 1, also known as Flt-1, binds secreted PlGF with high affinity, triggering activation of phosphoinositide 3-kinase-Akt, mitogen-activated protein kinase, and Jak/signal transducer and activator of transcription signaling pathways. These biologic cascades are heavily involved in angiogenic signaling. Through direct reciprocal action on endothelial cells, PlGF acts to regulate endothelial cell migration and proliferation. A main stimulus for PlGF secretion is tissue hypoxia, which triggers collateral vessel formation in ischemic tissues through the action of hypoxia-inducible factor 1α (Carmeliet et al., 2001).

**Figure 1:**
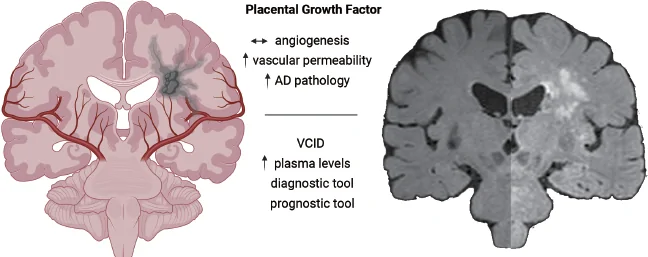
Placental growth factor regulates cerebral microvascular permeability and drives white matter lesions in vascular cognitive impairment and dementia (VCID). Normal levels of placental growth factor (PlGF) are necessary for brain vascular angiogenesis and the regulation of brain vascular permeability. When abnormal, PlGF increases brain vascular permeability and may synergize with Alzheimer’s disease (AD) pathologies. Elevated plasma levels of PlGF serve as diagnostic and prognostic biomarkers of vascular cognitive impairment and dementia associated with white matter hyperintensities on magnetic resonance imaging (MRI) (right) and can differentiate from normal MRI patterns (left). Created with BioRender.com.

In the developing brain, PlGF is critical for the formation of the cerebral circulation. In PlGF knockout mice, the cerebral circulation fails to develop, leading to prenatal mortality. In the adult brain, PlGF is primarily upregulated in pathophysiologic conditions. In models of brain ischemia, PlGF levels rise rapidly after stroke. In patients with advanced cerebral small vessel disease, there is a compendium of evidence supporting reduced brain perfusion, reduced microvascular density, and poor collateral blood flow circulation. Thus, CSVD is marked by a strong, low-grade chronic ischemic stimulus driving PlGF secretion. Additionally, a key role for PlGF appears to be regulation of vascular permeability which relates directly to cerebrovascular disease including stroke and VCID. PlGF is recognized to increase vascular permeability, which is essential for its role in the highly permeable placenta, but detrimental to its role in the brain, where permeability needs to be tightly controlled (**Figure 1**). A variety of imaging and pathologic evidence indicates that a key upstream pathologic event in CSVD and VCID is altered cerebrovascular permeability. This blood–brain barrier disruption can be measured *in vivo* by gadolinium leakage across the blood–brain barrier on MRI or increased albumin gradient in the cerebrospinal fluid (CSF). On pathology, excess fibrinogen and other abundant serum proteins deposit within the interstitial spaces in deep white matter in the brains of patients with VCID and CSVD. These findings suggest not only that aberrant angiogenesis and dysregulated brain permeability occur in CSVD, but also that PlGF is a key player in regulating both pathogenic cascades relevant to VCID.

**Placental growth factor as a diagnostic biomarker for vascular contributions to cognitive impairment and dementia:** Given the strong association of PlGF with the fundamental pathologic processes driving CSVD, plasma and CSF levels of PlGF have recently been explored as biomarkers for VCID disease states. A majority of these biomarker studies have focused on the association of circulating levels of PlGF with white matter MRI abnormalities, including a higher burden of T2/FLAIR hyperintensities and altered tissue microstructure measured via DTI, as these are sine qua non hallmarks of CSVD. In the MarkVCID Consortium, aimed at identifying reproducible biomarkers of cerebrovascular disease, a multi-site observational cohort study design examined the diagnostic accuracy of PIGF. In a cohort of 335 subjects without clinical Alzheimer’s disease, plasma PlGF levels were diagnostically accurate in identifying subjects with mild cognitive impairment due to vascular disease (area under curve [AUC] = 0.74) and highly accurate in identifying subjects with vascular dementia (AUC = 0.89) (Hinman et al., 2023). Another study confirmed the relationship of PlGF with WMH by measuring CSF levels in participants without dementia in the Swedish BIOFINDER study (Gertje et al., 2023). For 495 cognitively unimpaired older adult participants and 247 patients with mild cognitive impairment, higher CSF PlGF was the only biomarker tested that was cross-sectionally associated with higher WMH burden, even after adjusting for amyloid β-protein status.

Because PlGF can drive both angiogenesis and regulate vascular permeability, a recent study led by our team examined the role of PlGF in regulating brain vascular permeability through an indirect DTI measure (Kern et al., 2025). Cerebral white matter free water fraction is a DTI metric that reflects the water content in the interstitial space and is associated with gadolinium-based permeability measures. This advanced neuroimaging measure is strongly associated with T2/FLAIR WMH and other CSVD imaging markers, although it is also sensitive to alterations in normal-appearing white matter, preceding the development of overt WMH. Higher free water fraction due to increased interstitial water content can reflect the downstream consequences of a leaky blood–brain barrier that progresses to myelin and neuroaxonal injury, ultimately emerging as visible white matter lesions. Using imaging data from MarkVCID subjects, we showed that cerebral white matter free water is associated with plasma levels of PlGF. Employing a mediation analysis, we were able to show that free water mediated 73% of the PIGF-WMH association and 26% of the PIGF-cognition link. These findings broadly support a hypothesis that PlGF is a key regulator of brain vascular permeability and that elevated levels are associated with aberrant neoangiogenic mechanisms. While the temporal dynamics between PlGF, free water, and cognition still need to be confirmed, we hypothesize that PlGF is an upstream marker for vascular-mediated white matter injury.

**Placental growth factor as a predictive biomarker for vascular contributions to cognitive impairment and dementia:** The predictive capacity of PlGF for WMH progression or cognitive decline may be more nuanced. In the Swedish BioFINDER study, higher PlGF was associated with less WMH progression over up to 6 years (Gertje et al., 2023). In another longitudinal aging cohort study (*n* = 435), higher PlGF was associated with WMH progression at older ages but inversely associated at younger ages < 65 years (Torres-Espin et al., 2024). From these data, one might speculate that early PlGF elevations may be protective, although further investigations in middle-aged cohorts are needed. Regardless of age group, higher PlGF was consistently associated with worse executive function. Critically, the effects of PlGF on brain atrophy demonstrated a significant degree of age- and sexual dimorphism, with higher levels of PlGF associated with less brain atrophy in women under the age of 75 compared to men, an effect that reverses for women over 75 years old (Torres-Espin et al., 2024). In a longitudinal Alzheimer’s disease (AD) autopsy study, 90 individuals had pre-mortem plasma levels of PlGF collected within 2 years of post-mortem neuropathological evaluation. In these cases, higher pre-mortem PlGF was associated with more severe neuropathological cerebral amyloid angiopathy, but was surprisingly not associated with cerebral arteriolosclerosis (Winder et al., 2023). While both pathologies are forms of CSVD, cerebral amyloid angiopathy commonly co-occurs with AD, and arteriolosclerosis is primarily driven by hypertension and other systemic vascular risk factors.

**Placental growth factor with comorbid neurodegenerative disease:** Vascular contributions to cognitive impairment and dementia do not occur in isolation, and several studies have identified PlGF as a key biomarker in the setting of comorbid AD pathology. A cross-sectional study of memory clinic patients from Singapore found that serum PlGF was elevated in 109 with AD, but not in 76 with cognitive impairment-no dementia, compared to 56 who were cognitively intact. PlGF was further elevated in AD patients with a higher burden of WMH or cerebral microbleeds and was associated with CSVD severity (Wu et al., 2024). Higher cerebrovascular disease burden is believed to accelerate clinical AD progression, so the role of PlGF as a candidate biomarker and potential mediator for this synergism warrants further investigation. The association between PlGF and neurodegenerative disease progression was studied in 317 cognitively unimpaired older adults from the Harvard Aging Brain Study by collecting plasma angiogenic factors at baseline (vascular endothelial growth factor A and PlGF) and serially measuring cognition, PET-amyloid burden, and PET-tau burden over 12 years (Yang et al., 2024). For participants with high baseline amyloid (pre-clinical AD), low vascular endothelial growth factor A and high PlGF were associated with accelerated tau accumulation and steeper cognitive decline. Furthermore, tau accumulation partially mediated 31% of the association between higher PlGF and cognitive decline, findings that were robust after adjusting for cardiovascular risk and WMH volume. These associations between PlGF and tau accumulation may also be relevant in diagnosing other neurodegenerative diseases, such as frontotemporal dementia (FTD), a primary tauopathy. Another study from the Swedish BioFINDER cohort measured PlGF in the CSF for 27 FTD patients, 50 healthy older controls, and 251 patients with other forms of mild cognitive impairment or dementia (Hansson et al., 2019). Compared to controls, CSF PIGF was higher in FTD patients and provided very high classification performance with an AUC of 0.996. When PlGF was added to an established biomarker combination of amyloid β-protein 42 and tau, the classification performance for distinguishing FTD from all other dementias improved from an AUC of 0.932 to 0.972. These results support the use of a combination of biomarkers for distinguishing the myriad pathologies that accelerate cognitive decline. The contribution of PlGF to neurodegenerative disease also implicates a role for potentially targetable angiogenic mechanisms in the development and progression of several forms of dementia.

**Next steps:** Despite the strong clinical evidence for PlGF as a mediator of aberrant angiogenesis and altered cerebrovascular permeability as key pathogenic processes in CSVD and VCID, there remains a disconnection between our fundamental understanding of PlGF biology in the brain and its utility as a biomarker. Ultimately, biomarkers can serve many different roles in clinical practice depending on their accessibility and performance characteristics. While high test sensitivity facilitates screening, and high specificity supports diagnosing diseases, high reproducibility and dynamic temporal resolution is useful for monitoring disease progression and treatment efficacy. While biologic relevance is critical, the precise mechanisms of good diagnostic biomarkers need not be fully understood. However, the ability of PIGF to reflect cerebrovascular pathology and cognitive decline suggested that it could also be a potential therapeutic target. This would require a deeper knowledge of PlGF signaling pathways in cerebral blood vessels and their longitudinal levels in patients at varying stages of VCID and AD. For now, PlGF exists as a signature of brain vascular injury with strong applications for the diagnosis of VCID. A longitudinal study to test whether PlGF can predict cognitive trajectories in a cognitively unimpaired cohort enriched with cerebrovascular risk factors would add further validity to the key role that aberrant angiogenesis plays in CSVD. Mechanistic studies of the precise role of PlGF on brain vasculature in the context of amyloid and tau pathology may also further drive an understanding of this critical molecule.

*This work was supported by the following grants: NS100608 and NS100614.*
